# Association Between Adipose Tissue Characteristics and Metabolic Flexibility in Humans: A Systematic Review

**DOI:** 10.3389/fnut.2021.744187

**Published:** 2021-12-03

**Authors:** Alice Glaves, Francisco Díaz-Castro, Javiera Farías, Rodrigo Ramírez-Romero, Jose E. Galgani, Rodrigo Fernández-Verdejo

**Affiliations:** ^1^Departamento de Nutrición, Diabetes y Metabolismo, Facultad de Medicina, Pontificia Universidad Católica de Chile, Santiago, Chile; ^2^Laboratorio de Investigación en Nutrición y Actividad Física (LABINAF), Instituto de Nutrición y Tecnología de los Alimentos (INTA), Universidad de Chile, Santiago, Chile; ^3^Carrera de Nutrición y Dietética, Departamento de Ciencias de la Salud, Facultad de Medicina, Pontificia Universidad Católica de Chile, Santiago, Chile; ^4^Laboratorio de Fisiología del Ejercicio y Metabolismo (LABFEM), Escuela de Kinesiología, Facultad de Medicina, Universidad Finis Terrae, Santiago, Chile

**Keywords:** body composition, respiratory quotient, metabolic health, obesity, fuel oxidation

## Abstract

Adipose tissue total amount, distribution, and phenotype influence metabolic health. This may be partially mediated by the metabolic effects that these adipose tissue characteristics exert on the nearby and distant tissues. Thus, adipose tissue may influence the capacity of cells, tissues, and the organism to adapt fuel oxidation to fuel availability, i.e., their metabolic flexibility (MetF). Our aim was to systematically review the evidence for an association between adipose tissue characteristics and MetF in response to metabolic challenges in human adults. We searched in PubMed (last search on September 4, 2021) for reports that measured adipose tissue characteristics (total amount, distribution, and phenotype) and MetF in response to metabolic challenges (as a change in respiratory quotient) in humans aged 18 to <65 years. Any study design was considered, and the risk of bias was assessed with a checklist for randomized and non-randomized studies. From 880 records identified, 22 remained for the analysis, 10 of them measured MetF in response to glucose plus insulin stimulation, nine in response to dietary challenges, and four in response to other challenges. Our main findings were that: (a) MetF to glucose plus insulin stimulation seems inversely associated with adipose tissue total amount, waist circumference, and visceral adipose tissue; and (b) MetF to dietary challenges does not seem associated with adipose tissue total amount or distribution. In conclusion, evidence suggests that adipose tissue may directly or indirectly influence MetF to glucose plus insulin stimulation, an effect probably explained by skeletal muscle insulin sensitivity.

**Systematic Review Registration:** PROSPERO [CRD42020167810].

## Introduction

Human adipose tissue has been extensively characterized. Studies have analyzed the total amount, distribution, and even the phenotype of adipocytes. Many of these characteristics have been associated with health. Elevated fat percentage, as an index of adipose tissue total amount, increases the incidence of cardiovascular disease (1), and also all-cause and cardiovascular disease mortality (2). Central fat distribution, which is assessed by the waist-to-hip ratio, increases the incidence of cardiovascular disease, even after adjustment for fat percentage (1). High percentages of fat in the trunk, but not in the legs, are associated with increased mortality from cardiovascular disease (2), which is partially explained by phenotypic differences in the adipocytes within those regions (3). Finally, the volume and activity of brown adipose tissue have been proposed to influence health, yet this is still controversial (4). Many of these associations may be partially mediated by the effect that adipose tissue exerts on other nearby and distant tissues.

Adipose tissue secretes adipokines that regulate fuel oxidation in other tissues. Adiponectin, for example, stimulates lipid oxidation, glucose uptake, and lactate production in skeletal muscle cells (5). Adipose tissue also releases fatty acids, and thus influences fuel availability in other tissues. Of note, the adipokines secreted, as well as the rate and profile of released fatty acids, differ among adipose tissue depots (6–8). Thus, by influencing fuel oxidation and availability, different adipose tissue characteristics may modulate (positively or negatively) metabolic flexibility (MetF), i.e., the capacity of cells, tissues, and the organism to adapt fuel oxidation to fuel availability (9). An impaired MetF has been proposed to appear at the early stages of metabolic disturbances (10) and has been associated with several metabolic disturbances (9). This suggests that adipose tissue characteristics may influence MetF, which subsequently affects metabolic health.

Evidence for an association between adipose tissue characteristics and MetF is somewhat scattered. In the study where the concept of MetF was coined, Kelley et al. (11) compared lean subjects vs. obese subjects. MetF was assessed in response to glucose plus insulin infusions (hereafter insulin stimulation) as the change in leg respiratory quotient (RQ = VCO_2_/VO_2_), an index of the relative oxidation of glucose and lipid. Subjects with obesity had a blunted increase in glucose oxidation when the exogenous glucose availability increased. This response reflected an impaired MetF in obesity. Subjects with obesity also had reduced lipid oxidation during fasting (i.e., high fasting RQ), which was considered another marker of impaired MetF, although fasting RQ by itself does not necessarily reflect an adaptation of fuel oxidation to fuel availability (9, 12). Therefore, in this seminal publication, high levels of total adipose tissue (i.e., obesity) were associated with an impaired MetF. A subsequent study directly analyzed the association of MetF with some adipose tissue characteristics, namely fat percentage and the size of subcutaneous adipocytes (13); the increase in whole-body RQ during insulin stimulation was considered as the marker of MetF [mostly skeletal muscle MetF (9)]. Therein, subjects with the highest fat percentage and the largest abdominal subcutaneous adipocytes had the lowest MetF, independent of BMI. These findings suggested that impaired MetF was associated with high amounts of adipose tissue and hypertrophic adipocytes. Since the capacity for hypertrophy characterizes trunk adipocytes (3), this capacity may be somehow related to metabolic disturbances. Notably, the study also showed that subjects with the highest MetF had the highest circulating adiponectin concentrations, thus suggesting that adiponectin influences (positively) MetF in skeletal muscle (13).

The aforementioned studies suggested an association between certain adipose tissue characteristics and MetF. Nevertheless, MetF was only assessed by measuring the RQ in response to insulin stimulation. There are currently many other metabolic challenges to measure MetF in humans, e.g., meal consumption, oral glucose, exercise, prolonged fasting (9, 10, 12, 14). RQ is commonly used to determine MetF in response to these challenges. As far as we know, the evidence including measurements of the various adipose tissue characteristics and MetF in response to different challenges has not been systematically summarized. Moreover, many of the challenges to measuring MetF had been used even before the MetF concept was coined. Thus, there may exist studies that, technically, measured MetF, but without referring to it as MetF. Therefore, we aimed to systematically review the evidence for an association between adipose tissue characteristics (total amount, distribution, and phenotype) and MetF in response to different metabolic challenges in human adults. This information will allow us to gain insight into the possible role of MetF in mediating the effects of adipose tissue on health.

## Methods

### Protocol and Registration

The systematic review protocol and the reporting methodology followed the PRISMA 2020 and SWiM guidelines (15, 16). Supplementary Table 1 and Supplementary Table 2 show the PRISMA and SWiM checklists, respectively. The protocol was prospectively registered in PROSPERO (CRD42020167810), and is publicly available at: https://www.crd.york.ac.uk/prospero/display_record.php?ID=CRD42020167810. Note that the search strategy registered in PROSPERO was updated to include the terms “metabolic inflexibility,” “men,” and “women.”

### Eligibility Criteria

The population was humans aged 18 to <65 years with any nutritional status, including athletes, people with reduced mobility, and patients afflicted with insulin resistance, metabolic syndrome, diabetes, kidney failure, hypertension, dyslipidemia, lipodystrophy, and anorexia or other mental illnesses. In reports that included a group not meeting these criteria (e.g., bariatric surgery), but another group meeting the criteria (e.g., control without surgery), only the group meeting the criteria was considered. The exposure was any of the following adipose tissue characteristics: (a) total amount in absolute (i.e., fat mass in kg) or relative (i.e., fat mass in %) values; (b) distribution, considered as either segmental (trunk, arm, gluteofemoral), anatomical [visceral (VAT), subcutaneous (SAT), intrathoracic], or bodily (gynoid, android); and (c) adipocyte phenotype, considered as either volume or cell type (white, brown, beige/brite). The primary outcome was a measure of MetF in response to metabolic challenges, operationalized as a change in RQ (ΔRQ). As a secondary outcome, we considered studies that measured MetF using other indicators, e.g., fasting RQ. All study designs were considered.

### Information Sources and Search

The search was conducted in PubMed, from its inception to September 4, 2021 (the final search was conducted on that day). We included reports in English or Spanish. The search strategy was as follows: (“Adipose Tissue”[Mesh] OR “Body Constitution”[Mesh] OR “Adipocytes”[Mesh] OR “Body Composition”[Mesh] OR “Gluteofemoral” OR “Torso”[Mesh] OR “Magnetic Resonance Spectroscopy”[Mesh] OR “Tomography, X-Ray Computed”[Mesh] OR “Electric Impedance”[Mesh] OR “Absorptiometry, Photon”[Mesh] OR “Trunk” OR “Body Fat” OR “Fat mass”) AND (“Metabolic Flexibility” OR “Respiratory Quotient” OR “RQ” OR “Metabolic Inflexibility”) AND (Human[Mesh] OR “Men” OR “Women”).

### Study Selection, Data Collection, and Data Items

Two researchers (AG and RF-V) independently screened the title and abstract of all identified records. The same researchers then independently assessed full-text reports for eligibility. Discrepancies in the selection process were resolved through discussion. From 20 of the selected reports, two researchers (AG and FD-C) extracted the same data, independently, as a pilot to standardize the collection process. Then, half of the remaining reports were assigned to each of these researchers (AG and FD-C) for data extraction. The data from the 22 reports included in our main analyses were additionally extracted by another researcher independently (RF-V), i.e., these reports were doubly-extracted. The following data items were collected: reference, study design, sample characteristics (size, age, sex, health condition, and nutritional status), adipose tissue characteristics along with the method of measurement, and MetF along with the method of measurement.

### Risk of Bias in Individual Studies

We used the checklist by Downs and Black (17) to assess the methodological quality of individual reports. The checklist includes 27 questions and was designed to be applied to randomized and non-randomized studies. The score assigned to question no. 27 (statistical power) was modified as previously reported (18): 1 point was assigned if the report had sample size calculation or 0 points if it did not have. We expressed the methodological quality as the points obtained relative to the maximum score of 28 points. From two selected studies, methodological quality was assessed by two researchers (AG and FD-C), independently, as a pilot to standardize the process. The pilot was repeated by another two couples of researchers (AG and JF; AG and RR-R). Then, the remaining reports were distributed between these four researchers (AG, FD-C, JF, and RR-R) to assess the methodological quality. The researchers met every other week to solve doubts.

### Analysis

Since the focus of most of the studies was not the association between adipose tissue and MetF, we explored this association using a simple approach. From selected reports, we compared MetF between adults with different adipose tissue characteristics, or compared changes in MetF and adipose tissue characteristics before vs. after interventions. If adipose tissue characteristics were associated with MetF, subjects with (statistically significant) different adipose tissue characteristics would be expected to have (statistically significant) different MetF. Further, interventions that modify (statistically significant) adipose tissue characteristics would be expected to also modify (statistically significant) MetF. Based on this premise, we used a vote counting methodology to estimate whether the evidence suggests an association and its directionality. This methodology was deemed appropriate considering the variability in the reports.

## Results

### Included Studies

**Figure 1:**
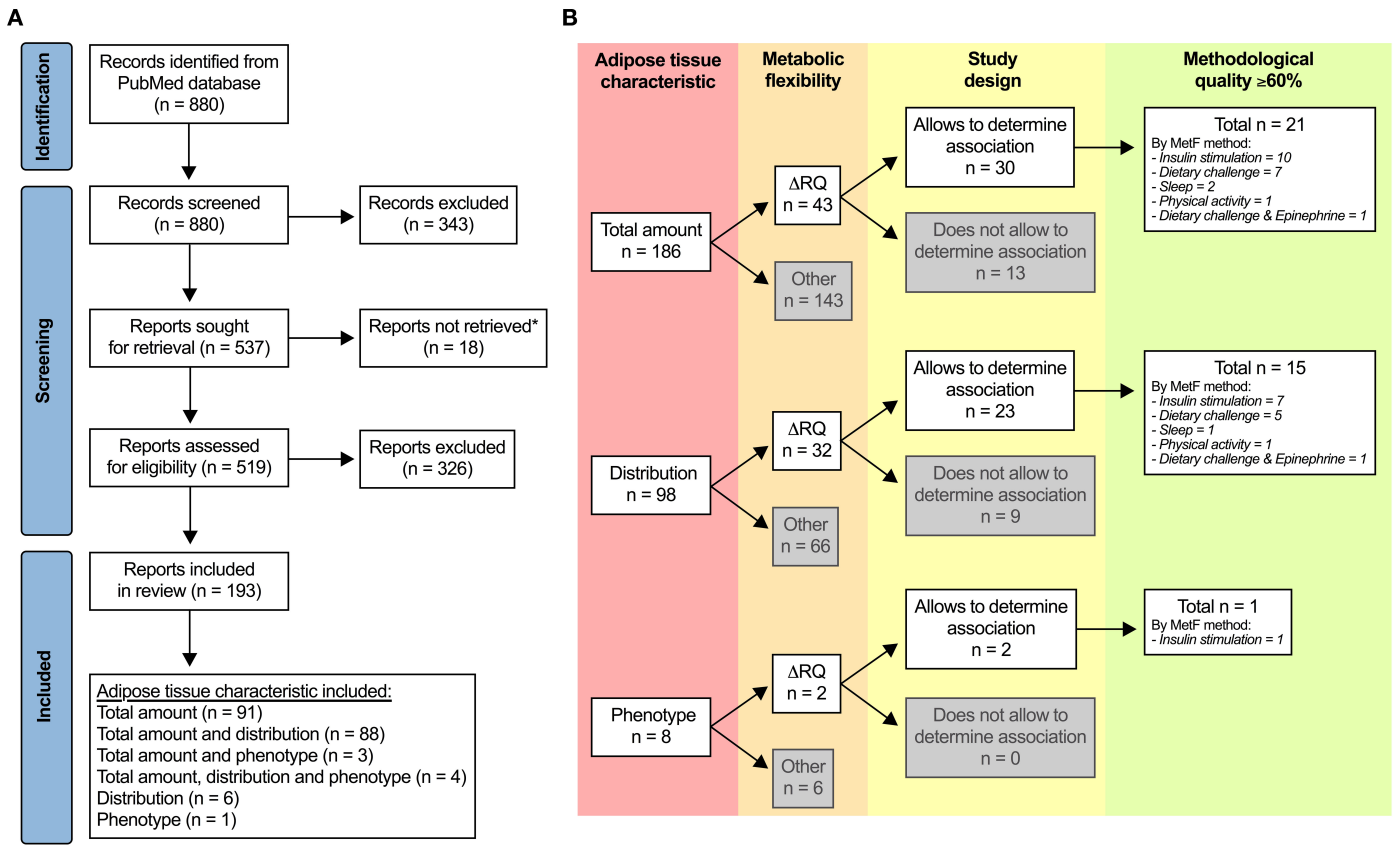
Selection of studies. **(A)** PRISMA flow diagram. *Unavailable for download in PubMed, ResearchGate (including a request to the authors), or other online websites. **(B)** Additional criteria to identify the most relevant evidence regarding the association between adipose tissue characteristics and metabolic flexibility (MetF). ΔRQ, change in respiratory quotient in response to a metabolic challenge.

Figure 1A shows the PRISMA flow diagram. From 880 records identified, 193 met the eligibility criteria. Ninety-six percent of the reports (*n* = 186) had measurements of adipose tissue total amount, either alone or together with distribution and/or phenotype. The reports had wide variability in terms of the index of MetF, study design, methodological quality, among other variables. This situation prompted us to add additional eligibility criteria than those we registered in PROSPERO to consider only the best evidence.

Figure 1B summarizes the additional eligibility process. First, we included only reports that used ΔRQ in response to metabolic challenges as the index of MetF, which was our primary outcome. Second, we included only reports with designs that allowed us to explore the association between adipose tissue characteristics and MetF, i.e., reports wherein: (a) the association between the variables was tested; (b) there were two or more groups that differed in at least one of the variables; and (c) there was a change in at least one of the variables after an intervention. Third, we included only reports with ≥60% of the score in the assessment of methodological quality. After applying these criteria, 22 reports remained for the analysis: 21 of them for adipose tissue total amount (19–39), 15 of them for adipose tissue distribution (22–24, 27–29, 31–38, 40), and one of them for adipose tissue phenotype [(37); Figure 1B]. The methodological quality was 60–69% in 16 reports, 70–79% in three reports, 80–89% in two reports, and ≥90% in one report.

Note that the included reports measured MetF using different methods (Figure 1B). Each method challenges the capacity of different tissues to adapt the oxidation to the availability of exogenous and/or endogenous substrates (9). Consequently, the association between adipose tissue characteristics and MetF may depend on the method used to measure MetF. For clarity, we, therefore, grouped and analyzed the reports according to the method used to measure MetF. Table 1 summarizes the reports that used insulin stimulation (*n* = 10), Table 2 those that used dietary challenges (*n* = 9), and Table 3 those that used other challenges (sleep *n* = 2, physical activity *n* = 1, epinephrine *n* = 1). The reports within each table were sorted based on the method to measure MetF (only Table 3), then study design, and finally the health status of the subjects. The following subsections describe these reports in the same order as they are presented in the tables. Finally, Supplementary Table 3 summarizes the reports excluded due to methodological quality, and Supplementary Table 4 those excluded due to other reasons.

**Table 1:** Summary of reports that measured MetF in response to glucose plus insulin infusions (ΔRQ = insulin-stimulated RQ – fasting RQ).

**References/Methodological quality**	**Design**	**Method for adipose tissue^**&**^**	**Subjects**			**MetF**	**Adipose tissue**		**Interpretation**
			**Groups**	**Men/Women (*n*)**	**Age (year)**		**Total amount**	**Distribution**	
Amador et al. (19)/82%	8-week exercise training	FM by DXA	Healthy	10/0	22.5 [SEM 0.8]	Pre 0.07 [SEM 0.01]; post 0.09 [SEM 0.02]	Pre 29.3% [SEM 2.1]; 28.0% [SEM 2.0] ^*^vs. pre. Pre 23.9 [SEM 2.7] kg; post 23.9 [SEM 2.7] kg	WHR: pre 0.89 [SEM 0.01]; post 0.87 [SEM 0.01]	Training decreased FM (%) in both groups, but MetF did not change; suggests no association. Not possible to determine association for WHR
			Healthy with family history of T2D	10/0	23.4 [SEM 0.9]	Pre 0.08 [0.02]; post 0.11 [SEM 0.02]	Pre 31.6% [SEM 2.0]; post 30.0% [SEM 1.9] ^*^vs. pre. Pre 24.2 [SEM 1.9] kg; post 23.2 [SEM 1.8] kg	WHR: pre 0.86 [SEM 0.01]; post 0.84 [0.01]	
Bak et al. (20)/61%	Cross-sectional	FM by DXA	Healthy NW	9/0	24 [range 21–33]	Fasting RQ 0.82 [*SD* 0.04]; insulin RQ 0.85 [*SD* 0.04] *vs. Fasting	10.2 [range 7.4–14.0] kg	–	OB had higher FM, but lower MetF. Suggests association
			Healthy OB	9/0	24 [range 21–35]	Fasting RQ 0.80 [*SD* 0.03]; insulin RQ 0.81 [*SD* 0.03]	41.1 [range 34.1–54.7] ^***^vs. NW	–	
Adamska et al. (31)/64%	Cross-sectional	FM by BIA	Healthy NW	0/22	24.3 [*SD* 5.8]	0.08 [*SD* 0.08]	24.1% [*SD* 7.1]	WC: 72.3 [*SD* 6.0] cm	OW/OB had higher FM and WC, but lower MetF. Suggests association
			Healthy OW/OB	0/26	25.1 [*SD* 5.6]	0.01 [*SD* 0.06] ^*^vs. NW	39.4% [*SD* 8.2] ^*^vs. NW	WC: 95.6 [*SD* 11.9] cm ^*^vs. NW	
Adamska et al. (33)/64%	Cross-sectional	FM by BIA	Healthy NW	0/14	26.6 [*SD* 6.3]	0.06 [*SD* 0.09]	23.7% [*SD* 4.6]	WC: 73.9 [*SD* 5.8] cm	OW/OB had higher FM and WC, but lower MetF. Suggests association
			Healthy OW/OB	0/16	26.9 [*SD* 6.7]	0.001 [*SD* 0.05] ^**^vs. NW	39.7% [*SD* 8.8] ^**^vs. NW	WC: 98.1 [*SD* 13.9] cm ^**^vs. NW	
Adamska et al. (34)/61%	Cross-sectional	FM by BIA	Healthy NW	6/19	25.1 [*SD* 5.3]	0.05 [*SD* 0.05]	23.2% [*SD* 6.6]	WC: 74.2 [*SD* 6.1] cm	OB had higher FM and WC, but lower MetF. Suggests association
			Healthy OW	3/9	25.0 [*SD* 5.3]	0.02 [*SD* 0.05]	30.4% [*SD* 7.5]	WC: 85.3 [*SD* 5.9] cm ^*^vs. NW	
			Healthy OB	3/13	28.3 [*SD* 7.9]	−0.04 [*SD* 0.05] ^*^vs. NW	42.9% [*SD* 10.7] ^*^vs. NW and OW	WC: 104.1 [*SD* 6.9] cm ^*^vs. NW and OW	
Ukropcova et al. (38)/68%	Cross-sectional	FM by DXA; VAT by CT	Healthy NW/OW/OB	34/0	22.3 [*SD* 3.3]	0.098 [*SD* 0.049]	16.8 [*SD* 6.2] kg, 20.2% [*SD* 5.8]	VAT: 1.9 [*SD* 1.2] kg	MetF was inversely associated with FM (kg: *r* = −0.32*; %: *r* = −0.40*), and VAT (*r* = −0.35*). Suggests association
			Healthy NW/OW/OB with family history of T2D	16/0	22.3 [*SD* 2.1]	0.077 [*SD* 0.034]	17.0 [*SD* 8.7] kg, 20.3% [*SD* 7.5]	VAT: 2.1 [*SD* 1.3] kg	
Ukropcova et al. (39)/61%	Cross-sectional	FM by DXA	Healthy NW/OW/OB	16/0	22.4 [*SD* 3.6]	0.074 [*SD* 0.037]	15.89 [*SD* 6.8] kg, 19.7% [*SD* 6.8]	WC: 82.5 [*SD* 9.7] cm	FM (%) was inversely associated with MetF (*r* = −0.51*); suggests association. No analysis for WC
Sparks et al. (37)/61%	Cross-sectional	FM by DXA; VAT by CT; SAT not reported	Healthy NW/OW/OB men	56/0	22.6 [*SD* 3.2]	0.09 [*SD* 0.04]	20.3% [*SD* 6.5]	WHR: 0.87 [*SD* 0.07]. VAT: 2.1 [*SD* 1.3] kg. SAT: 4.9 [*SD* 0.4] kg	Groups had different FM, WHR, VAT, SAT, and MetF; suggests association. VAT was inversely associated with MetF in men (*R*^2^ = 0.13^**^) and women (*R*^2^ = 0.19, *P* = 0.05); suggests association
			Healthy NW/OW/OB women	0/22	22.7 [*SD* 3.4]	0.14 [*SD* 0.04] ^**^vs. men	32.7% [*SD* 5.4] ^**^vs. men	WHR: 0.78 [*SD* 0.07] ^**^vs. men. VAT: 1.3 [*SD* 0.8] kg ^**^vs. men. SAT: 7.1 [*SD* 0.6] kg ^**^vs. men	
Chomentowski et al. (35)/64%	Cross-sectional	FM by DXA	Insulin-sensitive NW	3/9	47.0 [SEM 2.1]	0.15 [SEM 0.02]	21.2 [SEM 1.3] kg, 32.6% [SEM 2.0]	WC: 81.8 [SEM 1.7] cm	Groups with higher FM and WC had lower MetF. Suggests association
			Insulin-resistant non-diabetic	5/12	44.0 [SEM 1.7]	0.07 [SEM 0.01] ^**^vs. insulin-sensitive NW	39.8 [SEM 1.6] kg ^*^vs. insulin-sensitive NW, 43.8% [SEM 1.8] ^**^vs. insulin-sensitive NW	WC: 110.7 [SEM 2.8] cm ^**^vs. insulin-sensitive NW	
			T2D	4/7	44.0 [SEM 2.7]	0.05 [SEM 0.01] ^**^vs. insulin-sensitive NW	38.2 [SEM 2.5] kg ^**^vs. insulin-sensitive NW, 38.9% [SEM 2.8]	WC: 109.4 [SEM 2.4] cm ^**^vs. insulin-sensitive NW	
Karczewska-Kupczewska et al. (36)/71%	Cross-sectional	FM by BIA	Healthy NW	0/24	24.1 [*SD* 4.8]	0.06 [*SD* 0.06]	25.56% [*SD* 7.66]	WC: 71.04 [*SD* 6.12] cm	Groups had different FM and WC, but similar MetF. Suggests no association
			Anorexia nervosa	0/21	22.4 [*SD* 5.2]	0.05 [*SD* 0.08]	12.93% [*SD* 4.15] ^*^vs. NW	WC: 61.05 [*SD* 3.55] cm ^*^vs. NW	

*Data are mean with standard deviation (SD) or standard error of the mean (SEM), frequencies or percentages*.

& *The method to measure WC or WHR was assumed as tape measure in all reports, because that is the standard procedure. ΔRQ, change in respiratory quotient; BIA, bioelectric impedance; CT, computed tomography scanning; DXA, dual-energy X-ray absorptiometry; FM, fat mass; MetF, metabolic flexibility; NW, normal-weight or lean; OB, obesity; OW, overweight; SAT, subcutaneous adipose tissue; T2D, type 2 diabetes; VAT, visceral adipose tissue; WHR, waist-to-hip ratio; WC, waist circumference*.

* *P < 0.05*,

** *P < 0.01, and*

*** *P < 0.001*.

**Table 2:** Summary of reports that measured MetF in response to dietary challenges.

**References/Methodological quality**	**Design**	**ΔRQ as**	**Method for adipose tissue^**&**^**	**Subjects**			**MetF**	**Adipose tissue**		**Interpretation**
				**Groups**	**Men/Women (*n*)**	**Age (year)**		**Total amount**	**Distribution**	
Lightowler et al. (21)/93%	12-week energy-reduced diet	Post-prandial – fasting	FM by air-displacement plethysmography	Healthy OW/OB, sucrose-supplemented diet	5/20	41.2 [*SD* 12.0]	Pre 0.07 [*SD* 0.05]; post 0.07 [*SD* 0.03]	Pre 40.1% [*SD* 6.2]; post 39.3% [*SD* 6.6]	WC: pre 90.7 [*SD* 7.5] cm; post 89.1 [*SD* 7.5] cm	Not possible to determine association for FM or WC
				Healthy OW/OB, isomaltulose-supplemented diet	4/21	40.2 [*SD* 12.0]	Pre 0.04 [*SD* 0.05]; post 0.06 [*SD* 0.05]	Pre 38.9% [*SD* 5.7]; post 37.0% [*SD* 6.9] ^*^vs. pre	WC: pre 91.4 [*SD* 10.2] cm; post 87.9 [*SD* 7.3] cm	FM decreased, but MetF did not change; suggests no association. Not possible to determine association for WC
Rudwill et al. (24)/71%	21-day bed rest in control or protein supplemented conditions (cross-over)	Max – min (over 420 min post-prandial)	FM by DXA; SAT, VAT, calf fat, and liver fat by MRI	Healthy NW, control condition	9/0	31.0 [SEM 2.1]	MetF decreased similarly in control and protein supplemented	Pre 18.6 [SEM 1.3] kg, 23.8% [SEM 1.1]; post 18.0 [SEM 1.1] kg, 23.5% [SEM 1.0]	SAT: pre 8,574 [SEM 980] px; post 8,049 [SEM 842] px ^*^vs. pre. VAT: pre 3,878 [SEM 549] px; post 3,538 [SEM 543] px. Calf fat: pre 4.5% [SEM 0.1]; post 4.8% [SEM 0.2] ^*^vs. pre. Liver fat: pre 3.2% [SEM 0.2]; post 2.8% [SEM 0.2] ^**^vs. pre	The decrease in SAT and the increase in calf fat were accompanied by decreases in MetF; suggests association. FM, VAT, and liver fat did not change as MetF; suggests no association
				Healthy NW, protein supplemented condition				Pre 17.7 [SEM 1.1] kg, 22.9% [SEM 1.0]; post 18.1 [SEM 1.1] kg, 23.5% [SEM 1.0]	SAT: pre 8,887 [SEM 1,058] px; post 7,646 [SEM 1,021] px ^*^vs. pre. VAT: pre 3,255 [SEM 444] px; post 3,478 [SEM 382] px. Calf fat: pre 4.6% [SEM 0.2]; post 4.8% [0.2] ^*^vs. pre. Liver fat: pre 2.9% [SEM 0.2]; post 2.9% [SEM 0.2]	
Kahlhöfer et al. (25)/68%	1-week overfeeding, 3-week energy restriction, and 2-week refeeding	iAUC after oral glucose	FM by MRI	Healthy NW/OW, 65% energy as CHO, and refeed with high (*n* = 8) or low (*n* = 8) glycemic CHO	16/0	24.2 [*SD* 3.2]	MetF did not change during refeeding (high glycemic CHO: −0.14 [*SD* 0.20]; low glycemic CHO: −0.13 [*SD* 0.14])	FM increased during refeeding (high glycemic CHO: 1.7 [*SD* 0.6] kg; low glycemic CHO: 1.0 [*SD* 0.4] kg)	–	The increases in FM during refeeding were not accompanied by changes in MetF. Suggests no association
				Healthy NW/OW, 50% energy as CHO, and refeed with high (*n* = 8) or low (*n* = 8) glycemic CHO	16/0	26.8 [*SD* 4.1]	MetF did not change during refeeding (high glycemic CHO: 0.02 [*SD* 0.18]; low glycemic CHO: −0.03 [*SD* 0.21])	FM increased during refeeding (high glycemic CHO: 1.1 [*SD* 0.7] kg; low glycemic CHO: 1.0 [*SD* 0.6] kg)	–	
Bergouignan et al. (26)/61%	1-month detraining	Variance following two consecutive meals	FM by DLW	Trained NW healthy	9/0	23.6 [SEM 1.1]	Interventions that reduced physical activity (detraining and bed rest), decreased MetF	Pre 10.5 [SEM 1.2] kg; post 11.4 [SEM 1.5] kg. Pre 14.5% [SEM 1.4]; post 15.8% [SEM 1.8] ^*^vs. pre	–	In interventions that decreased physical activity, the change in FM was not consistent with the change in MetF. Suggests no association
	1-month bed rest			Normally-active NW healthy	0/8	33.9 [SEM 0.8]		Pre 14.8 [SEM 3.7] kg; post 14.3 [SEM 1.3] kg. Pre 26.4% [SEM 5.5]; post 27.0% [SEM 1.9]	–	
				Normally-active NW healthy with exercise countermeasure	0/8	33.1 [SEM 0.9]		Pre 14.5 [SEM 3.2] kg; post 13.0 [SEM 1.3] kg ^*^vs. pre. Pre 25.0% [SEM 5.0]; post 23.2% [SEM 2.1] ^*^vs. pre	–	
	2-month exercise training			Sedentary NW healthy	10/0	27.2 [SEM 2.9]	Interventions that increased physical activity (training), did not change MetF	Pre 17.9 [SEM 1.9] kg; post 17.0 [SEM 1.7] kg. Pre 22.9% [SEM 1.8]; post 21.9% [SEM 1.7]	–	In interventions that increased physical activity, there was no change in FM or MetF. Not possible to determine association
				Sedentary OW healthy	9/0	29.4 [SEM 1.5]		Pre: 31.2 [SEM 1.5] kg; post 30.5 [SEM 1.5] kg. Pre 31.8% [SEM 1.0]; post 31.1% [SEM 1.0]	–	
Assaad et al. (40)/68%	Cross-sectional	Post-prandial – fasting	–	Healthy NW	8/0	23.5 [SEM 1.4]	No difference between groups up to 120 min post-prandial	–	WC: 93.9 [SEM 2.0] cm	Groups had different WC, but similar MetF. Suggests no association
				Healthy OB	7/0	22.7 [SEM 1.2]		–	WC: 118.9 [SEM 3.5] cm ^*^vs. NW	
Huda et al. (23)/82%	Cross-sectional	Post-prandial – fasting	Not reported	Healthy NW/OW	5/4	39.2 [SEM 4.2]	Post-prandial RQ increased*	23.6% [SEM 3.0]	WC: 77.8 [SEM 5.0] cm	OB had higher FM and WC, but lower MetF. Suggests association
				Healthy OB	3/6	40.2 [SEM 1.9]	Post-prandial RQ did not change	50.4% [SEM 1.6] ^*^vs. NW/OW	WC: 146.0 [SEM 10.6] cm ^*^vs. NW/OW	
Purtell et al. (22)/64%	Cross-sectional	Post-prandial – fasting	FM and abdominal FM by DXA	Healthy NW	5/5	28.8 [95%CI 26.2–31.4]	No difference between groups	14.6 [95%CI 10.8–18.5] kg, 24.3% [95%CI 17.7–31.0]	WHR: 0.79 [95%CI 0.74–0.84]. Abdominal FM: 1.03 [95%CI 0.82–1.24] kg, 24.9% [95%CI 19.7–29.8]	Groups had different FM, WHR, and abdominal FM, but similar MetF. Suggests no association
				OB with (*n* = 2) or without (*n* = 10) T2D	7/5	32.3 [95%CI 26.9–37.6]		40.3 [95%CI 32.9–47.8] kg ^*^vs. NW, 41.7% [95%CI 35.3–48.2] ^*^vs. NW	WHR: 0.90 [95%CI 0.85–0.95] ^*^vs. NW. Abdominal FM: 3.37 [95%CI 2.79–3.96] kg ^*^vs. NW, 46.3% [95%CI 41.5–51.0] ^*^vs. NW	
Bergouignan et al. (27)/61%	4-day LFD, and 4-day HFD	24 h LFD – 24 h HFD	Not reported	Healthy NW	4/6	30 [*SD* 8]	24 h RQ LFD 0.90 [SEM 0.01]; 24 h RQ HFD 0.84 [SEM 0.01] ^***^vs. LFD	17.6 [*SD* 4.6] kg, 26.6% [*SD* 6.5]	WC: 78.3 [*SD* 7.4] cm	Groups had different FM and WC, but similar MetF. Suggests no association
				Healthy OB	5/4	37 [*SD* 7]	24 h RQ LFD 0.90 [SEM 0.01]; 24 h RQ HFD 0.83 [SEM 0.01] ^***^vs. LFD	42.2 [*SD* 8.9] kg ^*^vs. NW, 39.5% [*SD* 3.3] ^*^vs. NW	WC: 107.9 [*SD* 16.4] cm ^*^vs. NW	
Berk et al. (28)/61%	1-week LFD, and 1-week HFD (cross-over)	LFD – HFD	FM by DXA; SAT and VAT by MRI	Healthy NW/OW/OB African-American	0/21	32.8 [*SD* 7.4]	LFD RQ 0.862 [*SD* 0.02]; HFD RQ 0.849 [*SD* 0.01]	29.3 [*SD* 14.2] kg, 35.3% [*SD* 10.6]	WHR: 0.84 [*SD* 0.09]; VAT: 60 [*SD* 44] cm^2^; SAT: 216 [*SD* 111] cm^2^	Groups had similar FM, WHR, VAT, and SAT, but different MetF. Suggests no association
				Healthy NW/OW/OB Caucasian	0/21	34.9 [*SD* 6.9]	LFD RQ 0.872 [*SD* 0.02]; HFD RQ 0.818 [*SD* 0.01] ^**^vs. LFD	29.1 [*SD* 15.6] kg, 36.5% [*SD* 11.9]	WHR: 0.83 [*SD* 0.12]; VAT: 68 [*SD* 36] cm^2^; SAT 205 [*SD* 99] cm^2^	

*Data are mean with standard deviation (SD) or standard error of the mean (SEM), frequencies or percentages*.

& *The method to measure WC or WHR was assumed as tape measure in all reports, because that is the standard procedure. ΔRQ, change in respiratory quotient; 95%CI, 95% confidence intervals; CHO, carbohydrates; DLW, doubly labeled water; DXA, dual-energy X-ray absorptiometry; FM, fat mass; HFD, high-fat diet; iAUC, incremental area under the curve; LFD, low-fat diet; MetF, metabolic flexibility; MRI, magnetic resonance imaging; NW, normal-weight or lean; SAT, subcutaneous adipose tissue; OB, obesity; OW, overweight; T2D, type 2 diabetes; VAT, visceral adipose tissue; WHR, waist-to-hip ratio; WC, waist circumference*.

* *P < 0.05*,

** *P < 0.01, and*

** *P < 0.001.

**Table 3:** Summary of reports that measured MetF in response to sleep, physical activity, and epinephrine infusion.

**References/Methodological quality**	**Design**	**ΔRQ as**	**Method for adipose tissue^**&**^**	**Subjects**			**MetF**	**Adipose tissue**		**Interpretation**
				**Groups**	**Men/Women (*n*)**	**Age (year)**		**Total amount**	**Distribution**	
Mynatt et al. (29)/61%	Cross-sectional	24 h – sleep	Not reported	Metabolically inflexible	7/7	33.9 [SEM 13.0]	24 h RQ: 0.90 [SEM 0.03]; Sleep RQ: 0.90 [SEM 0.03]	32.1 [SEM 18.0] kg, 34.3% [SEM 12.1]	WC: 99.7 [SEM 22.7] cm	Groups had similar adipose tissue characteristics. Suggests no association
				Metabolically flexible	8/8	26.1 [SEM 6.3]	24 h RQ: 0.89 [SEM 0.06]; Sleep RQ: 0.84 [SEM 0.08] ^**^vs. inflexible	26.4 [SEM 23.7] kg, 28.2% [SEM 14.6]	WC: 93.9 [SEM 27.8] cm	
Rynders et al. (30)/64%	3-day eucaloric feeding and 3-day overfeeding	Awake – sleep	FM by DXA	Healthy NW/OW, OB-prone	8/14	28.5 [*SD* 2.6]	OB-resistant had higher MetF in response to overfeeding	18.4 [*SD* 6.0] kg	–	The difference in MetF remained after adjusting for FM. Suggests no association
				Healthy NW, OB-resistant	16/14	28.0 [*SD* 2.6]		11.9 [*SD* 3.0] kg ^**^vs. OB-prone	–	
Júdice et al. (32)/75%	Cross-sectional	Variance during three physical activities	FM and trunk fat by DXA	Healthy NW/OW/OB men	25/0	32.5 [*SD* 11.4]	0.008 [*SD* 0.005]	16.5 [*SD* 7.3] kg, 20.7% [7.9]	Trunk fat: 8.5 [*SD* 4.8] kg, 21.8% [*SD* 8.9]	MetF (adjusted for sex and age) was associated with FM (%) and trunk fat (%) in inverse regression, but not linear models. Suggests association
				Healthy NW/OW/OB women	0/25	38.0 [*SD* 15.7]	0.008 [*SD* 0.007]	20.8 [*SD* 8.6] kg, 33.1% [8.1] ^**^vs. men	Trunk fat: 9.4 [*SD* 5.2] kg, 30.7% [*SD* 10.7] ^**^vs. men	
Berk et al. (28)/61%	Cross-sectional	Post – pre epinephrine infusion	FM by densitometry; SAT and VAT by MRI	Healthy OW/OB African-American	0/9	38 [*SD* 7]	Pre 0.916 [*SD* 0.02]; post 0.896 [*SD* 0.03]	36.4 [*SD* 10.2] kg, 40.7% [*SD* 6.6]	WHR: 0.83 [*SD* 0.07]; VAT: 74 [*SD* 53] cm^2^; SAT: 320 [*SD* 150] cm^2^	Groups had similar FM, WHR, VAT, and SAT, but different MetF. Suggests no association
				Healthy OW/OB Caucasian	0/8	36 [*SD* 8]	Pre 0.939 [*SD* 0.02]; post 0.826 [*SD* 0.03] ^**^vs. pre	37.1 [*SD* 7.7] kg, 42.9% [*SD* 4.6]	WHR: 0.86 [*SD* 0.08]; VAT: 109 [*SD* 62] cm^2^; SAT: 339 [*SD* 142] cm^2^	

*Data are mean with standard deviation (SD) or standard error of the mean (SEM), frequencies or percentages*.

& *The method to measure WC was assumed as a tape measure in all reports because that is the standard procedure. ΔRQ, change in respiratory quotient; DXA, dual-energy X-ray absorptiometry; FM, fat mass; MetF, metabolic flexibility; MRI, magnetic resonance imaging; NW, normal-weight or lean; OB, obesity; OW, overweight; SAT, subcutaneous adipose tissue; VAT, visceral adipose tissue; WHR, waist-to-hip ratio; WC, waist circumference*.

** *P < 0.01*.

### MetF in Response to Insulin Stimulation

Only the report by Amador et al. (19) had a clinical trial design. Therein, healthy men with or without a family history of type 2 diabetes (T2D) were exercise-trained for 8 weeks. In both groups, the fat mass percentage decreased, but MetF did not change.

The remaining nine reports had a cross-sectional design. Four of them compared MetF between healthy subjects with different nutritional status (20, 31, 33, 34). Those reports showed that subjects with higher adipose tissue total amount or waist circumference had lower MetF. The reports by Ukropcova et al. (38, 39) included healthy subjects with or without a family history of T2D. Therein, adipose tissue total amount (38, 39) and VAT (38) were inversely associated with MetF. Sparks et al. (37) compared healthy men and women; therein, women had higher adipose tissue total amount and SAT, lower waist-to-hip ratio and VAT and a similar volume in the adipocytes from the abdominal subcutaneous adipose tissue (women 0.57 [*SD* 0.04] μl vs. men 0.60 [*SD* 0.02] μl). All this was accompanied by higher MetF in women, which was interpreted as a sexual dimorphism in MetF driven by adipose tissue characteristics. Importantly, they also found an inverse association between VAT and MetF in both sexes. Chomentowski et al. (35) compared insulin-sensitive, insulin-resistant, and subjects with T2D. Therein, groups with higher adipose tissue total amount and waist circumference (insulin-resistant and T2D) had lower MetF. Finally, Karczewska-Kupczewska et al. (36) compared healthy women with women with anorexia nervosa. The groups had different fat mass and waist circumference, but similar MetF.

In summary, eight out of ten reports suggested that adipose tissue total amount was inversely associated with MetF (20, 31, 33–35, 37–39). Four out of five reports suggested that waist circumference was associated inversely with MetF (31, 33–35). The only report that analyzed the waist-to-hip ratio also suggested an inverse association with MetF (37). Both reports analyzing VAT showed an inverse association with MetF (37, 38). The only report that analyzed SAT suggested a direct association with MetF (37). Finally, the only report analyzing adipocyte phenotype suggested no association between adipocyte volume and MetF (37).

### MetF in Response to Dietary Challenges

Four reports had a clinical trial design (21, 24–26). Lightowler et al. (21) analyzed healthy subjects exposed to an energy reduced diet supplemented with either sucrose or isomaltulose. After 12 weeks, the isomaltulose-supplemented diet reduced fat mass percentage but had no effect on MetF to a breakfast. Rudwill et al. (24) analyzed healthy men exposed to 21 days of bed rest. Adipose tissue total amount and VAT did not change, whereas SAT decreased, and calf fat increased. These responses were accompanied by decreases in MetF to a meal. In another report, Kahlhöfer et al. (25) exposed healthy men to a 1-week overfeeding, followed by a 3-week energy restriction, and a 2-week refeeding. Total adipose tissue increased during the refeeding phase, without changes in MetF to a 75-g glucose intake. Finally, Bergouignan et al. (26) analyzed the effects of changes in the level of physical activity through detraining, bed-rest, or training in men and women. In general, the changes in fat mass were accompanied by inconsistent changes, or no changes, in MetF to meals.

Three other reports had a cross-sectional design (22, 23, 40). Assaad et al. (40) found that healthy men with different waist circumferences had similar MetF to a meal. In contrast, Huda et al. (23) found that subjects with higher fat mass percentage and waist circumference had lower MetF to a breakfast. Purtell et al. (22) compared healthy subjects of normal weight vs. subjects with obesity (with or without T2D). Therein, subjects with obesity had higher fat mass, waist-to-hip ratio, and abdominal fat, but similar MetF to a breakfast, than the subjects with normal weight.

The last two reports analyzing MetF to dietary challenges exposed healthy subjects to low-fat and high-fat diets and measured MetF as the difference in RQ between diets (27, 28). Bergouignan et al. (27) showed that subjects with obesity had higher fat mass and waist circumference, but similar MetF, than subjects with normal weight. While Berk et al. (28) found that African–American and Caucasian women had similar fat mass, waist-to-hip ratio, VAT, and SAT, but different MetF.

In summary, seven out of eight reports suggested no association between adipose tissue total amount and MetF (21, 22, 24–28). Similarly, two out of three reports suggested no association between waist circumference and MetF (27, 40). Both reports analyzing the waist-to-hip ratio suggested no association with MetF either (22, 28). The only report that analyzed SAT suggested a direct association with MetF (24). Finally, the reports that analyzed VAT (24, 28) or abdominal fat (22) suggested no association with MetF.

### MetF in Response to Other Metabolic Challenges

Two reports measured MetF to sleep (29, 30). Mynatt et al. (29) showed that subjects deemed as metabolically flexible or inflexible had similar fat mass and waist circumference. Rynders et al. (30) compared healthy subjects of obesity-prone or obesity-resistant phenotypes. Obesity-resistant subjects had lower fat mass, and higher MetF; however, the differences in MetF were maintained after adjusting for fat mass.

Júdice et al. (32) compared MetF in response to different physical activities between healthy men and women. Men had lower fat mass percentage and trunk fat percentage, but similar MetF, than women. But notably, after adjusting for sex and age, MetF was associated with fat mass percentage and trunk fat percentage in an inverse regression model.

Finally, Berk et al. (28) measured MetF in response to an epinephrine infusion. Therein, African-American and Caucasian women had similar fat mass, waist-to-hip-ratio, VAT, and SAT, but different MetF.

In summary, reports suggest that adipose tissue total amount and waist circumference are not associated with MetF to sleep (29, 30). The only report analyzing MetF to physical activity suggested an inverse association with adipose tissue total amount and trunk fat (32). Finally, the only report that measured MetF to epinephrine suggested no association with adipose tissue total amount, waist-to-hip ratio, VAT, or SAT (28).

## Discussion

Adipose tissue total amount, distribution, and phenotype have been all associated with human health (1–4). The effect would be partially mediated by the release of adipokines and fatty acids that influence other tissues' functions, including their MetF (6–8, 13). If so, an association between adipose tissue characteristics and MetF is expected. To explore whether such association exists, we systematically gathered the evidence that measured adipose tissue characteristics and MetF in humans. We found that the associations depended on the method used to measure MetF. Our main findings were that: (a) MetF to insulin stimulation seems inversely associated with adipose tissue total amount, waist circumference, and VAT; and (b) MetF to dietary challenges does not seem to be associated with adipose tissue total amount or distribution. These results suggest that adipose tissue characteristics influence only certain determinants of whole-body MetF.

MetF has been defined in different ways, but the core idea remains the capacity to adapt fuel oxidation to fuel availability (9, 41–44). This definition has been operationalized in a wide diversity of methods. There is a certain agreement that the method should include a metabolic challenge requiring an adaptation in fuel oxidation (9, 41–44). The classical challenge is glucose plus insulin infusions during a euglycemic–hyperinsulinemic clamp, but other challenges have been also used (9). Since the RQ reflects the relative oxidation of glucose and lipid, the ΔRQ in response to the selected metabolic challenge has been often considered as the marker of MetF (9). Importantly, each challenge will test the capacity of specific tissues to adapt fuel oxidation to a change in the availability of different exogenous or endogenous substrates (9). Note that fasting RQ has been sometimes considered as a maker of MetF by itself; however, fasting RQ does not reflect an adaptation (i.e., a change) in fuel oxidation and depends on the preceding diet (45, 46). The same holds true for other measures that, by themselves, do not necessarily adhere to the concept of MetF (e.g., sleep RQ, exercise RQ, and absolute fat oxidation). Notably, we found over 100 reports wherein adipose tissue characteristics and any of these other putative markers of MetF were measured. The main characteristics of these reports are summarized in Supplementary Table 4. Nevertheless, given a large amount of evidence gathered, we focused only on the reports that measured ΔRQ and that had moderate-to-high methodological quality.

Several reports used insulin stimulation during a euglycemic–hyperinsulinemic clamp as the challenge to measure MetF. During the clamp, insulin stimulation is accompanied by infusions of glucose to maintain glycemia constant. Since high insulin infusions inhibit hepatic glucose production, the clamp mostly challenges the capacity of skeletal muscle to increase exogenous glucose oxidation (9). Any step from glucose uptake to mitochondrial oxidation will thus determine MetF to insulin stimulation (9, 47). In this context, most cross-sectional evidence suggested that adipose tissue total amount, waist circumference, and VAT were inversely associated with MetF. The agreement among these adipose tissue characteristics is expected considering their direct association (48). Thus, compared with lean subjects, those with higher adipose tissue or waist circumference showed lower MetF (20, 31, 33–35). Also, inverse associations of MetF with adipose tissue total amount (38, 39) and VAT (37, 38) were observed in reports that included subjects with a wide range of BMI. Only three reports suggested a different association pattern. First, Sparks et al. (37) found that women had higher adipose tissue total amount and MetF than men, but adipose tissue distribution seems to be more relevant when comparing between sexes. Indeed, the same report showed an inverse association between VAT and MetF in both men and women (37), and since women had lower VAT than men, VAT potentially explains the proposed sexual dimorphism in MetF. Second, Karczewska-Kupczewska et al. (36) found no difference in MetF between lean women and women with anorexia nervosa, thus suggesting that the association does not occur at very low adipose tissue. Finally, Amador et al. (19) showed that exercise training decreased fat mass, but had no effects on MetF. Note that this report was the only one with a clinical trial design. Perhaps, changes in adipose tissue total amount take some time to impact MetF. This may explain that the association between adipose tissue total amount and MetF to insulin stimulation mostly appears in cross-sectional comparisons of subjects with established nutritional statuses. Yet future studies are required to test these temporal associations. Note that only one study analyzed the association between adipose tissue phenotype (adipocytes volume) and MetF (37), which we considered insufficient to draw conclusions.

Regression analyses have shown that skeletal muscle insulin sensitivity is the major determinant of MetF to insulin stimulation, explaining ~50% of MetF variance (9, 49, 50). The rationale is that the higher the glucose uptake, the higher the intracellular glucose available for oxidation (9). Considering this, several pieces of evidence suggest that skeletal muscle insulin sensitivity explains the association of adipose tissue total amount, waist circumference, and VAT with MetF to insulin stimulation. First, subjects with high adipose tissue total amount and waist circumference had lower insulin sensitivity than their lean counterparts (31, 33–35). Second, insulin sensitivity was directly associated with MetF (38, 39). Third, insulin sensitivity was similar in lean women and women with anorexia nervosa, which were groups with different fat mass yet similar MetF (36). Fourth, Bak et al. (20) showed that men with normal weight had higher insulin sensitivity and MetF than men with obesity after a 12-h fast; however, after a 72-h fast, differences in insulin sensitivity vanished and essentially the same occurred with MetF. Fifth, abdominal obesity (i.e., high waist circumference and VAT) has been long known to associate directly with insulin resistance (51). Note that the factors explaining the remaining MetF variance are yet to be determined. Fasting RQ, race, and circulating triglycerides and fatty acids have been implicated, but seem to play a minor role (49, 50). Identifying these determinants may help to understand, for example, the observation that exercise training can increase skeletal muscle insulin sensitivity without affecting MetF (19).

Other set of reports analyzed MetF in response to dietary challenges. In these challenges, fuel availability is at the intake level (9). MetF, therefore, depends on every level from the digestive system to mitochondrial oxidation, and also on the hormonal (insulin) responses. Moreover, the relative influence of each tissue on substrate oxidation is unknown (9), and during challenges including mixed meals, glucose, lipid, and proteins are oxidized simultaneously. Thus, MetF to dietary challenges represents a more physiological challenge, yet depends on a complex interaction of many systems. In this context, most reports suggested that adipose tissue characteristics were not associated with MetF, independent of the study design (21, 22, 24–28, 40). There were two exceptions. First, Huda et al. (23) showed that subjects with obesity had higher fat mass and waist circumference, but lower MetF to a breakfast, than their lean counterparts. The reason for the discrepancy is unknown but may depend on factors such as the specific dietary challenge (European style breakfast), among others. Second, Rudwill et al. (24) found that a 21-day bed rest decreased SAT, and this was accompanied by decreases in MetF to a meal and by increases in calf fat. This agrees with our recent observations of higher SAT in subjects with high vs. low MetF to a 75-g glucose intake (14). Together, this evidence suggests that SAT may regulate MetF to dietary challenges by storing fatty acids and thus preventing their ectopic accumulation. This could protect subjects from developing peripheral insulin resistance, but future studies should test this hypothesis.

With our search strategy and eligibility criteria, we identified few reports that measured MetF to other challenges. Two reports measured MetF in response to sleep, as the change from either 24-h RQ (29) or awake RQ (30) to sleep RQ. This method challenges the capacity of tissues to increase the oxidation of endogenous lipids. Thus, a larger drop in RQ during sleep indicates higher MetF (9). In this context, MetF depends on the capacity of adipose tissue to release lipids, and of other tissues to uptake and oxidize those lipids. Both reports suggested no association between adipose tissue characteristics and MetF to sleep (29, 30). One report measured MetF as the RQ variance to daily living activities, thus challenging the capacity of skeletal muscle to adapt the oxidation of endogenous substrates to variable energy demands (32). Another report measured MetF to epinephrine stimulation, thus challenging the capacity of beta-adrenergic-sensitive tissues to increase lipid oxidation (28). Given the few evidence for these methods, it may be too speculative to draw conclusions. Nevertheless, the study by Júdice et al. (32) revealed a fact worth considering. They observed that fat mass and trunk fat were not linearly associated with MetF; instead, an inverse regression model better fitted the associations. This suggests that models more complex than linear regression may describe the association between adipose tissue characteristics and MetF.

A limitation of our review process was that we only searched in PubMed, thus probably missing evidence from other databases. Note, however, that PubMed has been long recognized as an optimal tool for biomedical electronic research (52), and the most influential research on MetF is indexed therein [see (9, 41–44, 53) and their references]. Another limitation is that we focused only on reports in adult humans, thus excluding children, adolescents, and older adults, in whom several insightful studies have been conducted [see for example: (54–57)]. We also excluded *in vitro* studies because of the difficulty in operationalizing MetF in that model, but many of those studies have been described in previous narrative reviews (43, 44). The evidence included also has some limitations. First, although we included reports that measured adipose tissue characteristics and MetF, no study was specifically designed to test the association between these variables. The selection of participants, sample size, statistical analyses, among other factors may, thus, obscure the real association between adipose tissue characteristics and MetF. We used a rather simple strategy to ascertain whether the reports suggested an association (see section Analysis), and we used vote counting to summarize the information. But this strategy is sensitive to flaws, as revealed by the report by Rynders et al. (30). Therein, obesity-prone subjects had higher fat mass and lower MetF than obesity-resistant subjects, which may suggest an association; however, further analyses showed that the differences in MetF remained after adjusting for fat mass. Third, evidence suggesting an association between adipose tissue characteristics and MetF is mostly cross-sectional, therefore, a causal relationship cannot be inferred. The idea that the profile of adipokines and/or fatty acids plays an intermediary role between adipose tissue and MetF is thus only speculative at this point. Fourth, only one report remained for the analysis of adipose tissue phenotype (37), which we considered insufficient to draw conclusions. Finally, the search strategy was designed to identify reports that showed RQ values. Yet there may be reports mostly focused on other markers (e.g., lipid oxidation) that secondarily reported RQ and may have not been retrieved by the search engine [for example (58, 59)].

Our review also has some strengths worth mentioning. First, although several narrative reviews about MetF have been published (9, 41–44, 53), as far as we know, this is the first systematic review regarding MetF. Second, we only considered reports with moderate-to-high methodological quality to draw our conclusions; yet we presented the data for the reports with lower methodological quality as well (Supplementary Table 3). Finally, although we established additional criteria to select reports for analysis, all studies meeting our initial criteria are summarized in Supplementary Table 4. This table thus represents a valuable repository for researchers interested in other aspects of MetF.

In conclusion, we have shown that some adipose tissue characteristics are associated with MetF to insulin stimulation. Thus, evidence suggests that adipose tissue may directly or indirectly influence the capacity to adapt fuel oxidation to fuel availability in skeletal muscle, an effect most probably explained by insulin sensitivity. Our results have also highlighted the relevance of specifying the method used to measure MetF in future studies. This will allow us to better understand the role that MetF has on metabolic health along with the mechanisms involved.

## Data Availability Statement

The original contributions presented in the study are included in the article/Supplementary Material, further inquiries can be directed to the corresponding author/s.

## Author Contributions

AG conceived the study, acquired the data, processed the data, analyzed the data, and interpreted the data. FD-C, JF, and RR-R acquired the data and processed the data. JG conceived the study and interpreted the data. RF-V conceived the study, acquired the data, analyzed the data, interpreted the data, and drafted the manuscript. All the authors revised critically the manuscript and approved the final version.

## Funding

This study was funded by ANID/CONICYT FONDECYT Iniciación 11180361 to RF-V.

## Conflict of Interest

The authors declare that the research was conducted in the absence of any commercial or financial relationships that could be construed as a potential conflict of interest.

## Publisher's Note

All claims expressed in this article are solely those of the authors and do not necessarily represent those of their affiliated organizations, or those of the publisher, the editors and the reviewers. Any product that may be evaluated in this article, or claim that may be made by its manufacturer, is not guaranteed or endorsed by the publisher.
